# Multiple anti-tumor effects of Reparixin on thyroid cancer

**DOI:** 10.18632/oncotarget.16412

**Published:** 2017-03-21

**Authors:** Federica Liotti, Maria De Pizzol, Marcello Allegretti, Nella Prevete, Rosa Marina Melillo

**Affiliations:** ^1^ Dipartimento di Medicina Molecolare e Biotecnologie Mediche, University of Naples “Federico II”, Naples, Italy; ^2^ Dompé Farmaceutici S.p.A., L'Aquila, Italy; ^3^ Dipartimento di Scienze Mediche Traslazionali, University of Naples “Federico II”, Naples, Italy; ^4^ Istituto di Endocrinologia ed Oncologia Sperimentale del CNR “G. Salvatore”, Naples, Italy

**Keywords:** Reparixin, thyroid cancer, CXCR1, CXCR2

## Abstract

**Background:**

Expression of IL-8 and its receptors CXCR1 and CXCR2 is a common occurrence in human epithelial thyroid cancer (TC). In human TC samples, IL-8 expression is associated with tumor progression. IL-8 enhances proliferation, survival, motility, and leads to the maintenance of stemness features and tumor-initiating ability of TC cells. Here, we studied the effects of Reparixin (formerly Repertaxin), a small molecular weight CXCR1 and CXCR2 inhibitor, on the malignant phenotype of various TC cell lines.

**Results:**

Reparixin impaired the viability of epithelial thyroid cancerous cells, but not that of the non-malignant counterpart. Reparixin treatment significantly decreased TC cell survival, proliferation, Epithelial-to-Mesenchymal Transition (EMT) and stemness. CXCR1 and CXCR2 silencing abolished these effects. Reparixin sensitized TC cells to Docetaxel and Doxorubicin in culture. Used as single agent, Reparixin significantly inhibited TC cell tumorigenicity in immunodeficient mice. Finally, Reparixin potentiated the effects of Docetaxel on TC cell xenotransplants in mice.

**Materials and Methods:**

We assessed the effects of Reparixin on TC cell viability (by growth curves, BrdU incorporation, TUNEL assay), EMT (by RT-PCR, Flow Cytometry, Migration assays), stemness (by RT-PCR, Flow Cytometry, sphere-formation and self-renewal), and tumorigenicity (by xenotransplantation in nude mice).

**Conclusions:**

The present study suggests that Reparixin, both alone and in combination with classic chemotherapics, represents a novel potential therapeutic strategy for aggressive forms of TC.

## INTRODUCTION

Thyroid cancer (TC) is the most common endocrine malignancy. TC can arise from either the follicular or the medullary components of the thyroid gland. Carcinomas deriving from thyroid follicular epithelial cells include follicular (FTC), papillary (PTC), poorly differentiated (PDTC) and anaplastic thyroid carcinomas (ATC) [1]. FTC and PTC are also defined as differentiated TC (DTC), since they are treatable with surgery and I-131 ablation that eliminates primary tumor cells, and lymph-nodal and distant metastases. PDTC and ATC, instead, typically exhibit aggressive histologic features such as extrathyroidal extension, vascular invasion, and tumor necrosis [1]. Moreover, they are refractory to radioiodine and chemotherapics [2].

Interleukin (IL)-8 is a pro-inflammatory cytokine expressed by several cancer types, whose levels correlate with poor prognosis. IL-8 is the ligand for two related G-protein-coupled receptors, CXCR1 and CXCR2. These receptors, normally found on leukocytes, are also known to be expressed on epithelial normal and cancerous cells and on stromal cells of various cancer types [3]. IL-8 sustains cancer progression by promoting cancer cell proliferation, survival, Epithelial-to-Mesenchymal Transition (EMT), invasion, and angiogenesis [3]. Furthermore, IL-8 sustains the cancer stem-like cell (CSC) functional activities [4, 5], which have been related with chemoresistance and metastatic behavior of different cancer types [6]. For these reasons, the IL-8 signaling pathway has been recently proposed as an attractive target in many cancer types [4, 7].

IL-8 is considered an important marker of aggressiveness also in TC. Serum IL-8 levels correlate with TC stage and IL-8 staining in human TC samples correlates with the presence of lymph-nodal metastasis [8], thus indicating an association with tumor progression [9–11]. We recently found that TC-associated mast cells produce IL-8 [4, 12]. IL-8 was not only produced by tumor stroma, but also by TC cells [8]. Independently from its source, IL-8 sustains TC cell proliferation, survival, motility, induces EMT and, as we have recently shown, prompts TC stemness [4, 9].

In the present study, we assessed the effects of Reparixin (formerly Repertaxin), an allosteric, non-competitive, low molecular weight CXCR1-CXCR2 dual inhibitor [13], on several aspects of TC cell biology. In particular, we demonstrated that Reparixin impaired the viability of epithelial thyroid cancerous cells. Reparixin activity was specific for epithelial TC cells and undetectable in non-malignant epithelial thyroid cells. Reparixin significantly reduced functional and biochemical EMT and stemness features of TC cells both in basal conditions and upon IL-8 treatment. Docetaxel and Doxorubicin activities on TC cells both *in vitro* and *in vivo* were significantly potentiated by Reparixin. Finally, Reparixin, used as single agent, significantly inhibited TC cell tumorigenicity in immunodeficient mice [4, 14, 15],

These data indicate that Reparixin could be used to target IL-8 signaling for the treatment of aggressive forms of TC that do not respond to conventional therapies.

## RESULTS

### Reparixin affects thyroid cancerous cell proliferation and survival

**Figure 1:**
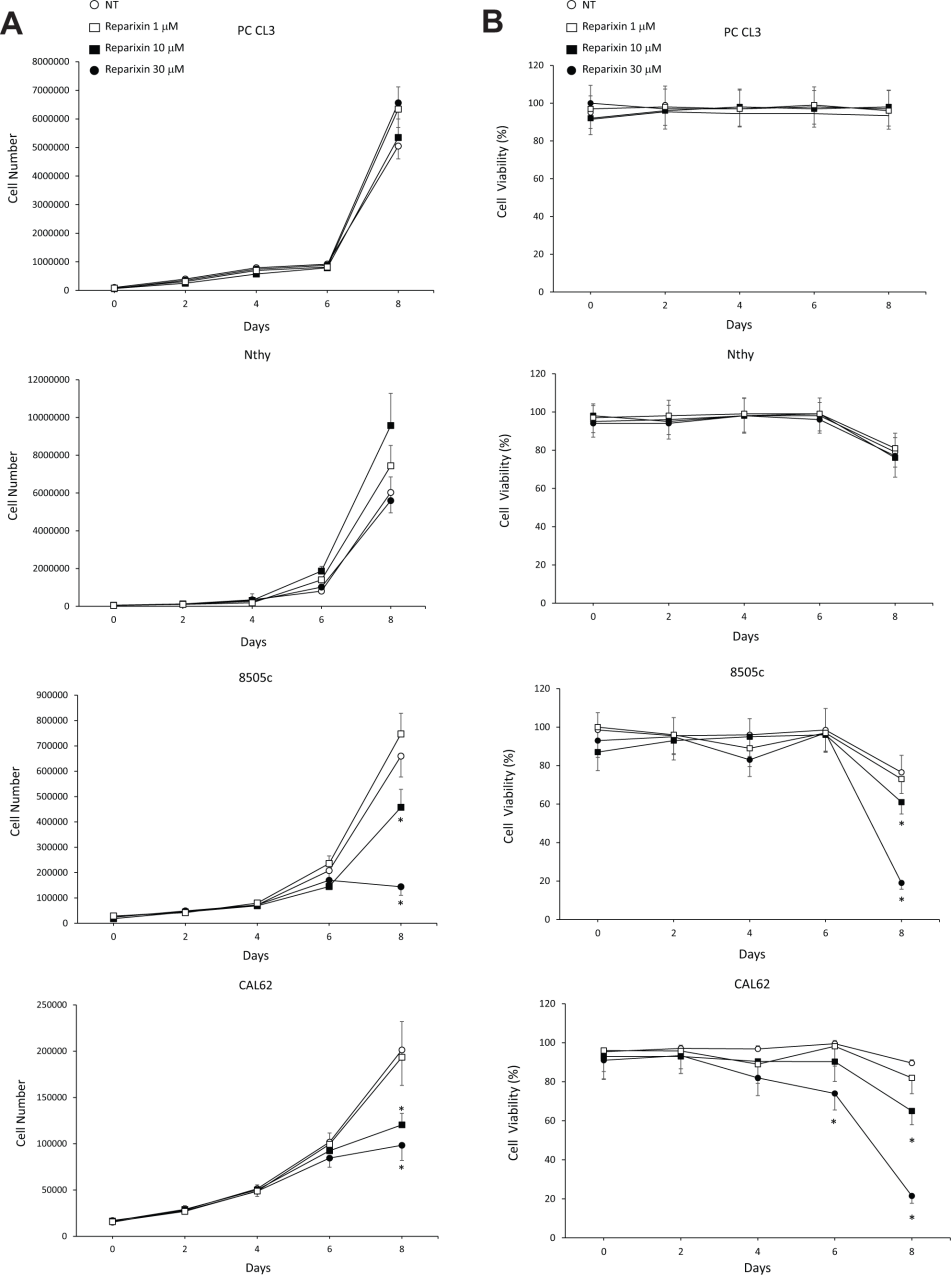
Reparixin affects TC cell proliferation (**A**) Growth curves of PC CL3, Nthy, 8505c and CAL62 cells in complete medium (DMEM 10% FBS) in the presence or absence of different concentrations of Reparixin (1 μM, 10 μM, 30 μM). The average results of at least 3 independent determinations were reported. *p < 0.05 compared to untreated cells (NT). (**B**) Cell viability evaluated by trypan blue of PC CL3, Nthy, 8505c and CAL62 cells treated or not with different concentrations of Reparixin (1 μM, 10 μM, 30 μM). The average results of at least 3 independent determinations were reported. The percent (%) of live cells was reported. *p < 0.05 compared to untreated cells (NT).

In order to determine the effects of Reparixin on thyroid epithelial cells, we selected PC CL3 (normal thyroid epithelial cell line derived from 18-month-old Fischer rats) [16] and Nthy-ori-3.1 (named Nthy throughout the text, human SV40 Large T-immortalized non tumorigenic human thyroid epithelial cell line) as representative of non-malignant thyroid cells [8]. 8505c, CAL62, and SW1736 cell lines (derived from human ATCs) were instead chosen as representative of undifferentiated and aggressive TC cells [8]. These ATC cell lines have been previously characterized for the expression of endogenous functional IL-8, CXCR1 and CXCR2 [8]. We measured the growth rate of these cell lines in complete medium (DMEM 10% FBS) in the presence or absence of different concentrations of Reparixin (0.1 μM, 1 μM, 10 μM, 30 μM). Growth curves, shown in Figure 1A, indicated that Reparixin inhibited 8505c and CAL62 cell growth in a dose-dependent manner after 8 days of culture. Similar results were obtained with SW1736 cells (data not shown). No significant effects were observed at 1 μM (Figure 1A) and 0.1 μM (data not shown) of Reparixin in all the cell lines tested. This effect was not observed in PC CL3 and Nthy cells, where a limited toxic effect was observed only after 10 days of treatment with 30 μM Reparixin (data not shown), being it significantly lower than that observed in TC cells.

We then evaluated the viability of PC CL3, Nthy, 8505c and CAL62 cell lines cultured in absence or in presence of Reparixin (0.1 μM, 1 μM, 10 μM, 30 μM) in complete medium at different time points, by using trypan blue staining of dead cells. 8505c and CAL62 cell viability significantly decreased after 8-days of exposure to Reparixin, in a dose-dependent fashion (Figure 1B). By contrast, we did not observe this effect in PC CL3 and Nthy cells cultured in the same conditions (Figure 1B) up to 10 days (data not shown). No significant effects of Reparixin on cell viability were observed at 0.1 μM (data not shown) neither on cancerous nor on normal cells. These results confirm that Reparixin in the micromolar range exerts its cytotoxic effects on cancerous thyroid epithelial cells but not on the non-malignant counterpart. Based on these results and accordingly to what has been observed in other experimental models [17, 18], we selected 30 μM as optimal concentration for further experiments.

**Figure 2:**
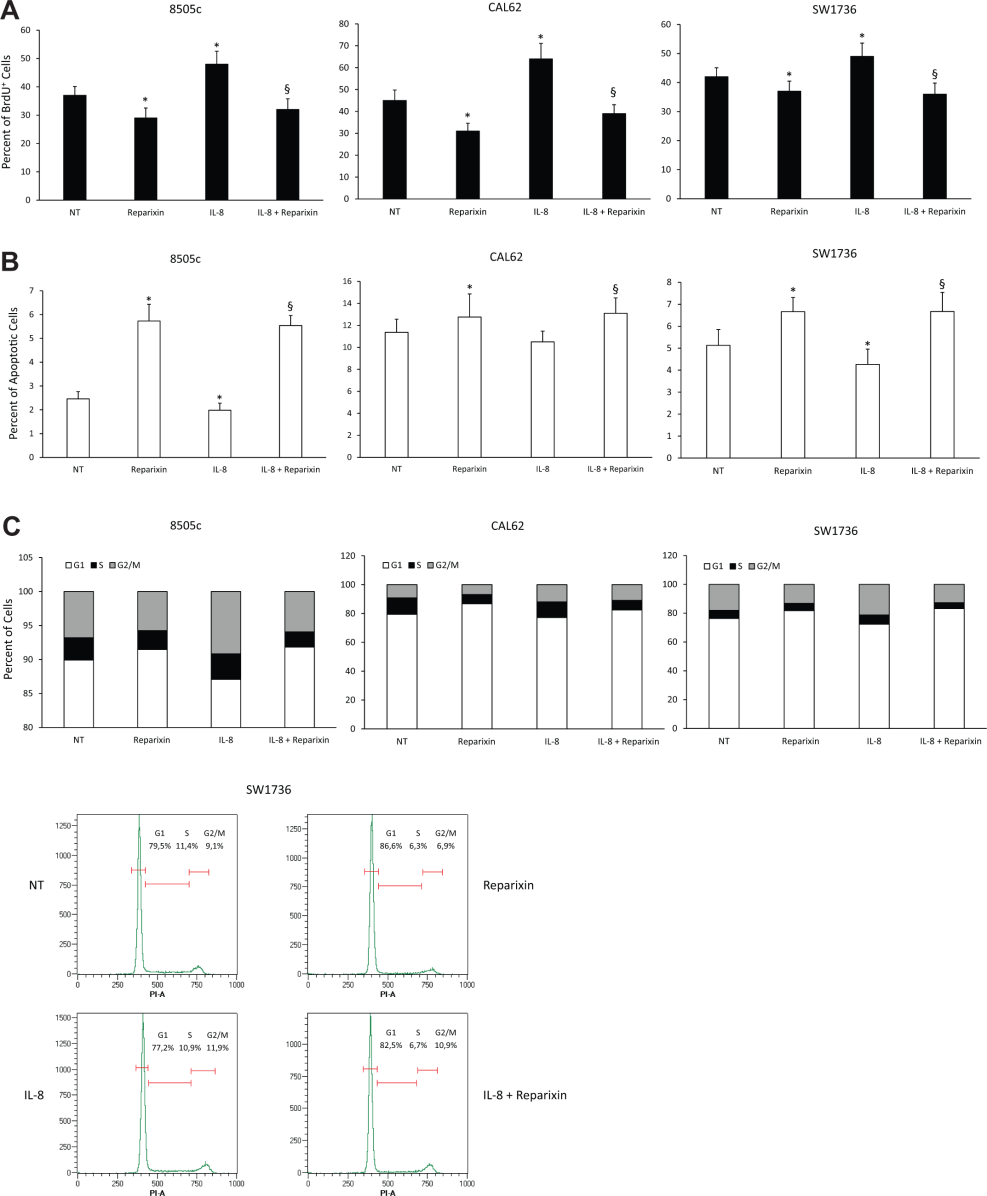
Reparixin inhibits TC cell proliferation by decreasing DNA synthesis and cell survival (**A**) BrdU incorporation at 24 h of 8505c, CAL62 and SW1736 treated with Reparixin (30 μM) both in the absence and presence of exogenous IL-8 (100 ng/ml). *p < 0.05 compared to untreated cells (NT); ^§^*p* < 0.05 compared to IL-8 treated cells. (**B**) Percent of apoptotic cells assessed by TUNEL reaction in 8505c, CAL62 and SW1736 treated with Reparixin (30 μM) both in the absence and presence for 24 h of exogenous IL-8 (100 ng/ml). *p < 0.05 compared to untreated cells (NT); ^§^*p* < 0.05 compared to IL-8 treated cells. (**C**) Cell cycle distribution of 8505c, CAL62 and SW1736 cells treated with Reparixin (30 μM), in the absence or in the presence of exogenous IL-8 (100 ng/ml), measured by Propidium Iodide (PI) staining by means of Flow Cytometry at 24 h. The percent of the cells distributed in G1, S, G2/M was indicated in each panel. A representative graph of SW1736 cell cycle was shown.

To dissect the cytotoxic effects of Reparixin on TC cells, we evaluated its influence on DNA synthesis, apoptosis and cell cycle in 8505c, CAL62 and SW1736 cell lines. We analyzed the levels of DNA synthesis by BrdU incorporation assay in serum-deprived cells treated or not with exogenous IL-8 (100 ng/ml) for 24 h, in the presence or absence of Reparixin (30 μM). Figure 2A shows that Reparixin treatment caused a significant reduction in the percentage of BrdU^+^ cells in all the analyzed cell lines, both in basal conditions and upon IL-8 treatment. To assess if Reparixin could induce TC cell apoptosis, we used a TUNEL assay. 8505c, CAL62 and SW1736 cells were serum-deprived, treated or not with Reparixin (30 μM), in the presence or absence of IL-8 (100 ng/ml) for 24 h. Reparixin significantly increased TC cell apoptotic rate in basal conditions. IL-8 treatment induced a limited but significant decrease in the apoptotic rate of 8505c and SW1736 cells, but Reparixin reverted this effect (Figure 2B).

To better define the mechanisms underlying Reparixin-mediated inhibition of TC cell growth, we measured the distribution of cells in the different phases of the cell cycle by flow cytometry. 8505c, CAL62 and SW1736 cells were treated or not with Reparixin (30 μM) and/or IL-8 (100 ng/ml) for 24 h in serum-deprived culture media. IL-8 caused a decrease in G1 and an accumulation in the S and G2/M phases of cell cycle of TC cells (Figure 2C). Reparixin treatment, both in the presence and in the absence of IL-8, inhibited cell cycle progression, as shown by the reduction in S-G2/M and the accumulation in G1 phases of 8505c and SW1736 cells (Figure 2C).

Taken together, these results demonstrated that Reparixin was able to reduce epithelial thyroid cancerous cell viability by increasing their apoptotic rate and by inhibiting DNA synthesis and cell cycle progression.

### Reparixin inhibits epithelial-to-mesenchymal transition (EMT) of TC cells

**Figure 3:**
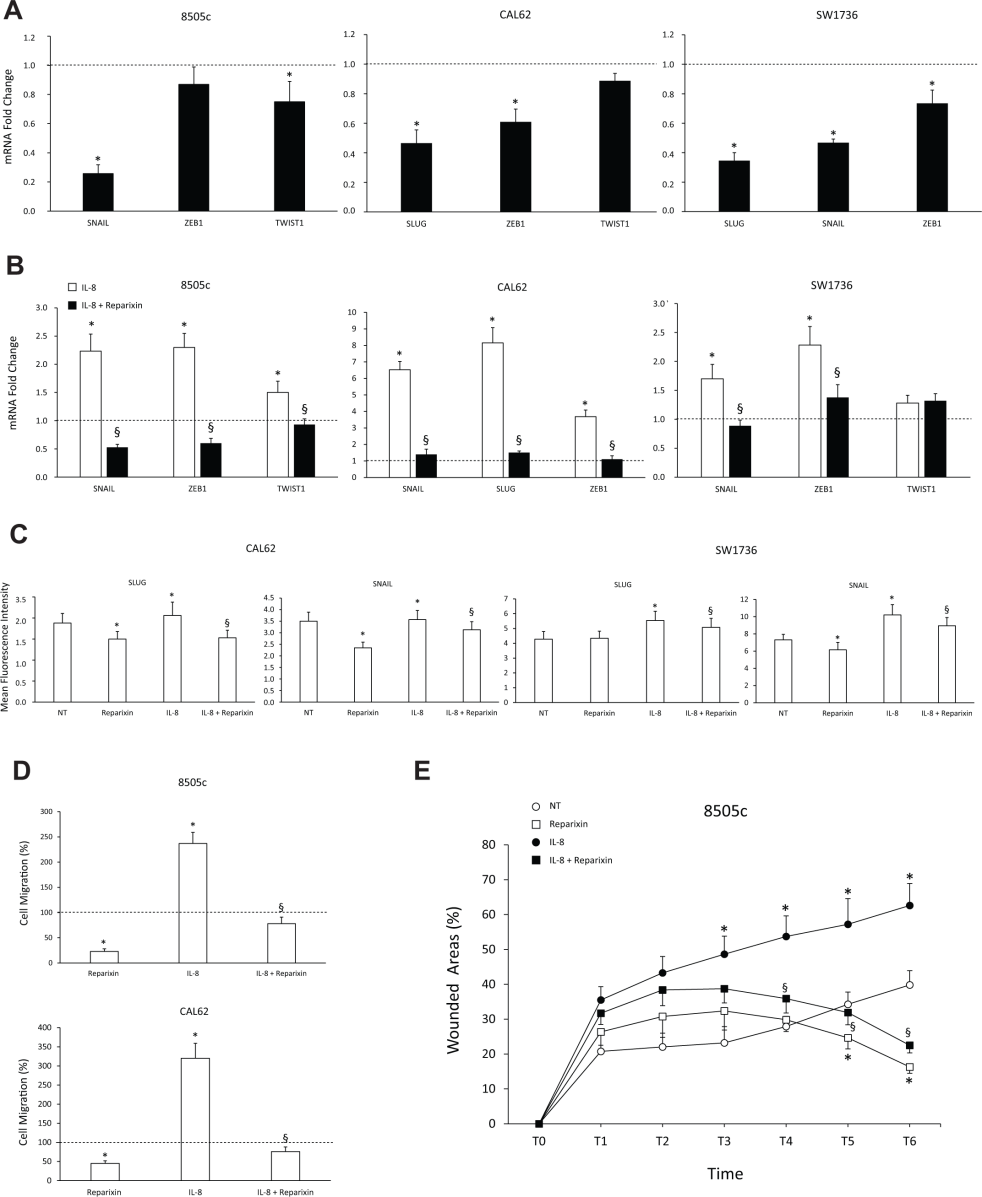
Reparixin inhibits the epithelial-to-mesenchymal transition (EMT) in TC cells (**A**–**B**) Effect of Reparixin (30 μM) on mRNA levels of the basal and IL-8 induced-EMT markers (SLUG, SNAIL, ZEB1, TWIST1) was evaluated by real time PCR in 8505c, CAL62 and SW1736 cells. Normalization to β-actin mRNA levels was used. For each target (x-axis), the expression levels were calculated relative to the expression levels in untreated cells, arbitrarily considered equal to 1 (dotted line). Experiments were performed in triplicate. *p < 0.05 compared to untreated cells (dotted line); ^§^*p* < 0.05 compared to IL-8 treated cells. (**C**) Cytofluorimetric evaluation of the indicated EMT markers levels in Reparixin (30 μM)-treated CAL62 and SW1736 cells, in the presence or absence of IL-8 (100 ng/ml). *p < 0.05 compared to untreated cells (NT); ^§^*p* < 0.05 compared to IL-8 treated cells. (**D**) Migration assay on Reparixin (30 μM)-treated 8505c and CAL62 cells, in presence or absence of IL-8 (100 ng/ml). The results were expressed as percentage of migrated cells compared to control (dotted line). *p < 0.05 compared to untreated cells (dotted line); ^§^*p* < 0.05 compared to IL-8 treated cells. (**E**) Wound-healing assays of 8505c cells treated with Reparixin (30 μM) in presence or absence of IL-8 (100 ng/ml) assessed in a time course experiment (0–18 h, every 3 h). Wound closure was measured by counting migrated cells in the wound area by high content analysis (Operetta, PerkinElmer). Experiments were performed in triplicate. *p < 0.05 compared to untreated cells (NT); ^§^*p* < 0.05 compared to IL-8 treated cells.

We previously demonstrated that IL-8 induces or potentiates the Epithelial-to-Mesenchymal Transition (EMT) in TC cells, thus increasing their invasive and migratory features [4]. In the present study, we examined the effects of Reparixin on biochemical and functional EMT of TC cells, by assessing EMT marker expression, cell invasion and migration. With this aim, we treated 8505c, CAL62 and SW1736 cells with Reparixin (30 μM) and evaluated mRNAs levels for EMT transcription factors (SNAIL1/SNAIL, SNAIL2/SLUG, TWIST1, ZEB1) by RT-PCR, either in basal conditions or upon 24 h stimulation with IL-8 (100 ng/ml). Reparixin significantly reduced the levels of mRNAs encoding various EMT transcription factors, both in basal conditions (Figure 3A) and upon IL-8 stimulation (Figure 3B) in all the cell lines analyzed. We also evaluated the effects of Reparixin on SNAIL and SLUG expression at the protein level by cytofluorimetric analysis in permeabilized CAL62 and SW1736 cells. Constitutive SLUG and SNAIL expression was downregulated by Reparixin (30 μM). As expected, treatment with IL-8 induced SLUG and SNAIL expression and co-treatment with Reparixin (30 μM) reverted this effect in both cell lines (Figure 3C). Similar results were obtained in 8505c cells (data not shown).

EMT-related activities include the acquisition of a motile phenotype [14]. To assess the activity of Reparixin on cell motility in TC cell lines, we used a Boyden chamber migration assay. Reparixin (30 μM) significantly reduced the migratory potential of 8505c and CAL62 cells both in the absence and in the presence of IL-8 (100 ng/ml) (Figure 3D). These observations were confirmed in a wound healing assay. In agreement with the above results, Reparixin-treated 8505c cells display lower migration ability when compared to untreated cells (Figure 3E). IL-8 treatment significantly increased the ability of 8505c cells to repair the wound, and Reparixin significantly inhibited it (Figure 3E).

Thus, Reparixin revealed a strong potential to inhibit both biochemical and functional EMT in TC cells.

### Reparixin inhibits TC cell stemness features

**Figure 4:**
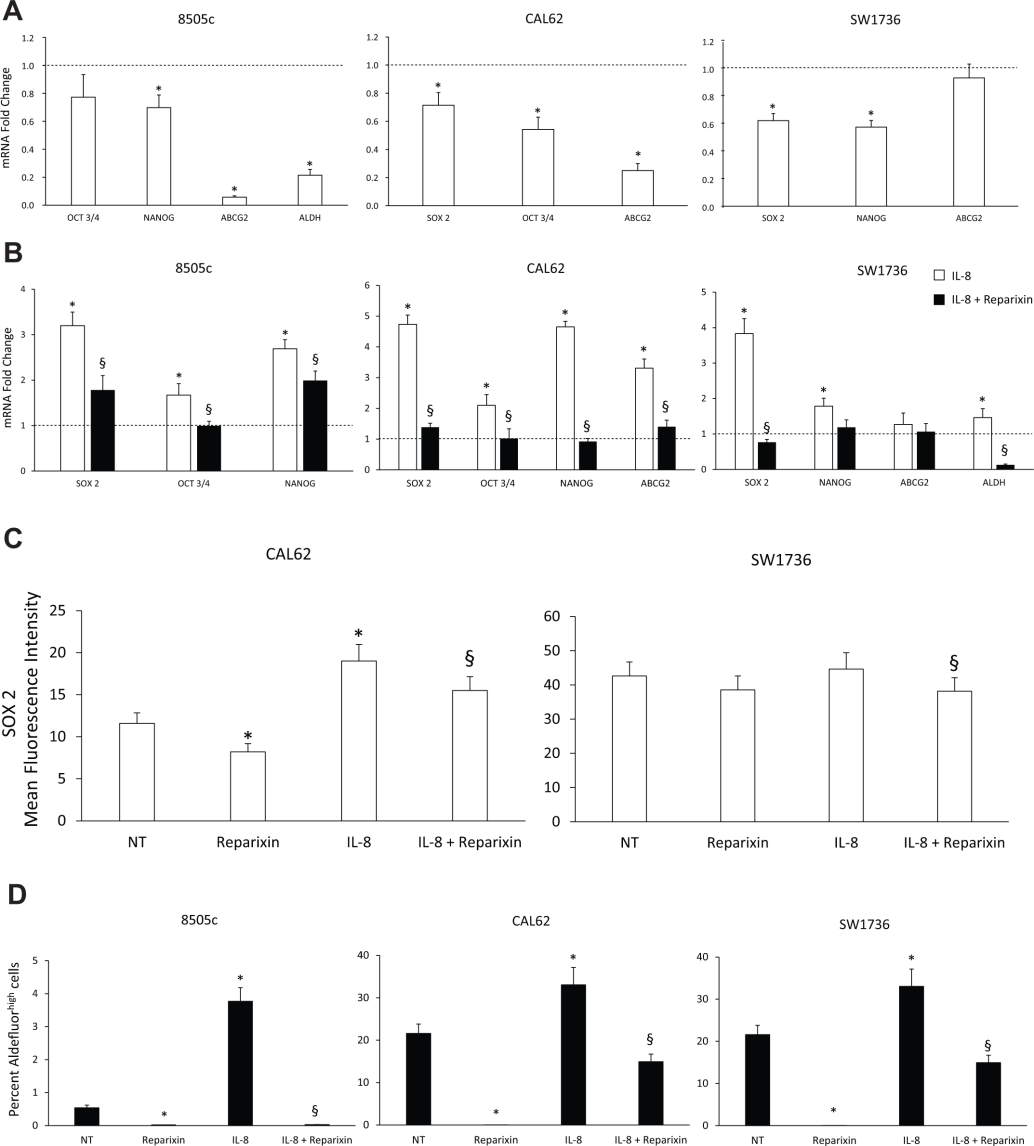
Reparixin inhibits stemness features of TC cells (**A**–**B**) Effect of Reparixin (30 μM) on mRNA levels of the basal and IL-8 induced-stemness markers (OCT3/4, NANOG, ABCG2, ALDH, SOX 2) was evaluated by real time PCR in 8505c, CAL62 and SW1736 cells. Normalization to β-actin mRNA levels was used. For each target (x-axis), the expression levels were calculated relative to the expression levels in untreated cells, arbitrarily considered equal to 1 (dotted line). Experiments were performed in triplicate. *p < 0.05 compared to untreated cells (dotted line); ^§^*p* < 0.05 compared to IL-8 treated cells. (**C**) Cytofluorimetric evaluation of SOX 2 stemness marker levels in Reparixin (30 μM)-treated CAL62 and SW1736 cells in the presence or absence of IL-8 (100 ng/ml). *p < 0.05 compared to untreated cells (NT); ^§^*p* < 0.05 compared to IL-8 treated cells. (**D**) Percent of Aldefluor^high^ cells in Reparixin (30 μM)-treated 8505c, CAL62 and SW1736 cells, in the presence or absence of IL-8 (100 ng/ml). *p < 0.05 compared to untreated cells (NT); ^§^*p* < 0.05 compared to IL-8 treated cells.

To assess the effects of Reparixin on stemness features, TC cell lines (8505c, CAL62, SW1736) were treated or not for 24 h with IL-8 (100 ng/ml), in the presence or absence of Reparixin (30 μM) and stemness marker expression (OCT3/4, SOX 2, ABCG2, NANOG, ALDH) was evaluated by real-time PCR. Reparixin significantly inhibited the expression of various stemness markers both in basal condition and upon IL-8 stimulation (Figure 4A and 4B). Cytofluorimetric analysis of the transcriptional factor SOX 2 in permeabilized CAL62 and SW1736 cells showed that Reparixin caused a significant reduction of this protein in untreated or IL-8-treated cells (Figure 4C). Consistently, Reparixin significantly reduced the percentage of 8505c, CAL62 and SW1736 displaying high Aldehyde Dehydrogenase enzymatic activity (ALDH^high^ cells), an established feature of thyroid CSCs [19], both in basal conditions and upon IL-8 treatment (Figure 4D).

**Figure 5:**
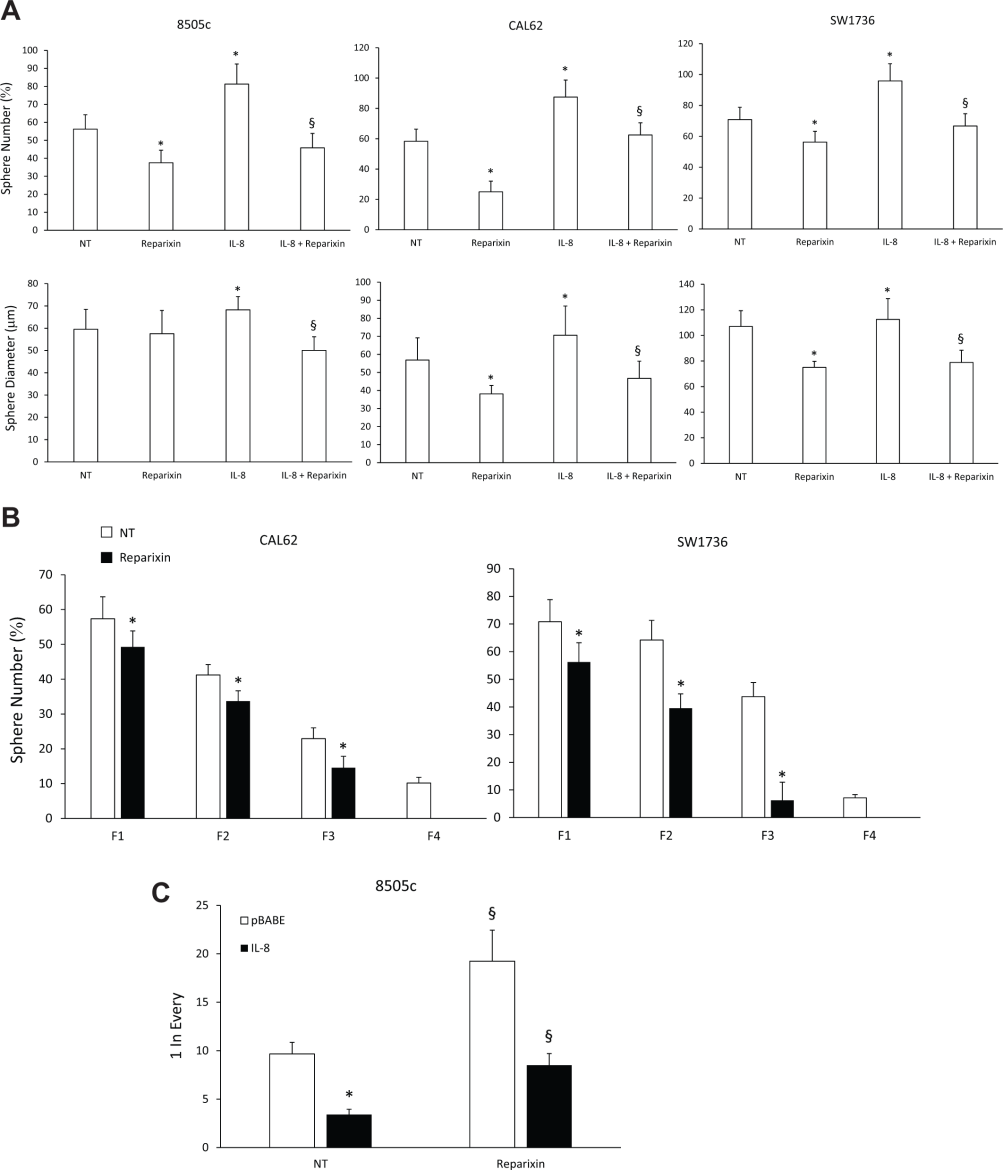
Reparixin reduces TC SCs functional activities (**A**) The sphere-forming efficiency of Reparixin (30 μM)-treated 8505c, CAL62 and SW1736 cells, in the presence or absence of exogenous IL-8 (100 ng/ml), evaluated as average sphere number and diameter. *p < 0.05 compared to untreated cells (NT); ^§^*p* < 0.05 compared to IL-8 treated cells. (**B**) Effect of Reparixin (30 μM) on self-renewal of CAL62 and SW1736 spheroids. Average number of formed spheres was shown. Generations were indicated as Fn. *p < 0.05 compared to untreated thyrospheres (NT). (**C**) Effect of Reparixin (30 μM) on the clonogenic potential of 8505c pBABE or IL-8 overexpressing spheroids. *P < 0.05 compared to 8505c pBABE; ^§^*p* < 0.05 compared to the relative not-treated (NT) spheroids.

To assess the effects of Reparixin on functions of TC stem cells (TC SCs), we performed a sphere-forming assay. We demonstrated that Reparixin (30 μM) significantly reduced the ability of 8505c, CAL62, and SW1736 cells to form spheroids (both in the presence and absence of IL-8), assessed as sphere number and/or diameter (Figure 5A). To assess Reparixin effects on self-renewal ability of thyrospheres, we used ATC cells grown in ultralow-adherent conditions for serial *in vitro* passages. CAL62 and SW1736 thyrospheres were serially passed through 4 generations (F1, F2, F3 and F4) in the presence or absence of Reparixin (30 μM) (Figure 5B). In comparison to untreated cells, Reparixin (30 μM) significantly reduced CAL62 and SW1736 sphere number in F1-F3, and completely abrogated their capacity to form spheres in F4 (Figure 5B). To assess the effect of Reparixin on the clonogenic capacity of TC cells, we performed a limiting dilution assay on 8505c cells grown as thyrospheres. Reparixin (30 μM) was able to significantly reduce the clonogenic potential of both IL-8 overexpressing and pBABE transfected cells (Figure 5C).

As already observed for breast CSCs [17], Reparixin significantly affected TC SCs by inhibiting their growth and perpetuation.

### Reparixin affects TC cell functions by acting on both CXCR1 and CXCR2 receptors

To evaluate whether Reparixin effects on TC cell viability and stemness could be ascribed to CXCR1 and/or CXCR2 antagonism, we used shCXCR1 and shCXCR2 8505c cells that we previously generated [8].

**Figure 6:**
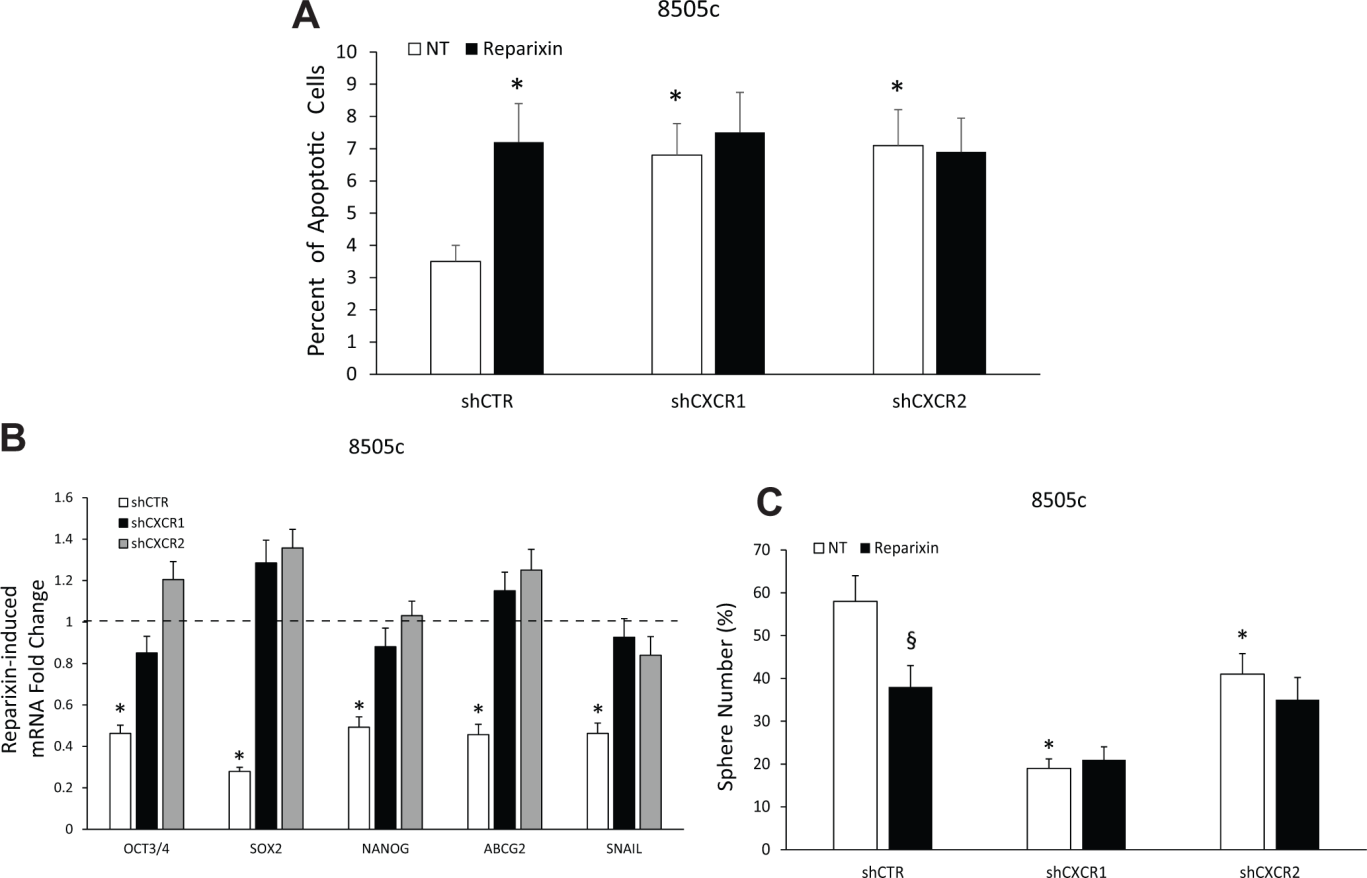
Reparixin effects on TC cells depend on both CXCR1 and CXCR2 (**A**) Percent of apoptotic cells assessed by TUNEL reaction in 8505c shCTR, shCXCR1 and shCXCR2 cells treated or not with Reparixin (30 μM) for 24 h. *p < 0.05 compared to untreated (NT) shCTR cells. (**B**) Effect of Reparixin (30 μM) on mRNA levels of the markers (OCT3/4, SOX 2, NANOG, ABCG2, SNAIL) was evaluated by real time PCR in 8505c shCTR, shCXCR1 and shCXCR2 cells. Normalization to β-actin mRNA levels was used. For each target (x-axis), the expression levels were calculated relative to the untreated cells, arbitrarily considered equal to 1 (dotted line). Experiments were performed in triplicate. *p < 0.05 compared to shCTR cells (dotted line). (**C**) Effect of Reparixin (30 μM) on sphere-forming efficiency of 8505c shCTR, shCXCR1, and shCXCR2, evaluated as average sphere number. *p < 0.05 compared to shCTR; ^§^*p* < 0.05 compared to the relative untreated control.

The involvement of CXCR1 and CXCR2 in the Reparixin-induced reduction of TC cell viability was evaluated by a TUNEL assay. Reparixin (30 μM) significantly increased the percentage of apoptotic cells in 8505c transfected with shCTR vector. This effect was completely abolished in cells silenced for CXCR1 or CXCR2 (Figure 6A). Similar experiments were performed to assess whether Reparixin effects on TC cell stemness could be abrogated in CXCR1- or CXCR2-silenced cells. Reparixin (30 μM) significantly reduced stemness (OCT3/4, SOX 2, NANOG, ABCG2) and EMT (SNAIL) marker expression in control cells, being this effect lost in shCXCR1 or shCXCR2 cell lines (Figure 6B). shCXCR1, and to a lesser extent shCXCR2, significantly reduced the ability of 8505c cells to form spheroids [8]. Reparixin (30 μM) significantly reduced sphere formation of shCTR cells. No significant effects of Reparixin were observed on shCXCR1 sphere number. Instead, a reduction in shCXCR2 thyrosphere number was observed upon Reparixin treatment, being this effect possibly due to Reparixin inhibition of CXCR1 in shCXCR2 cells (Figure 6C). However, due to the short half-life of Reparixin [17], its effect on shCXCR2 cells was not statistically significant.

These data demonstrated that both the reduction of viability and the inhibition of stemness caused by Reparixin in TC cells are linked to its ability to inhibit both CXCR1 and CXCR2.

### Reparixin reduces TC cell chemoresistance

**Figure 7:**
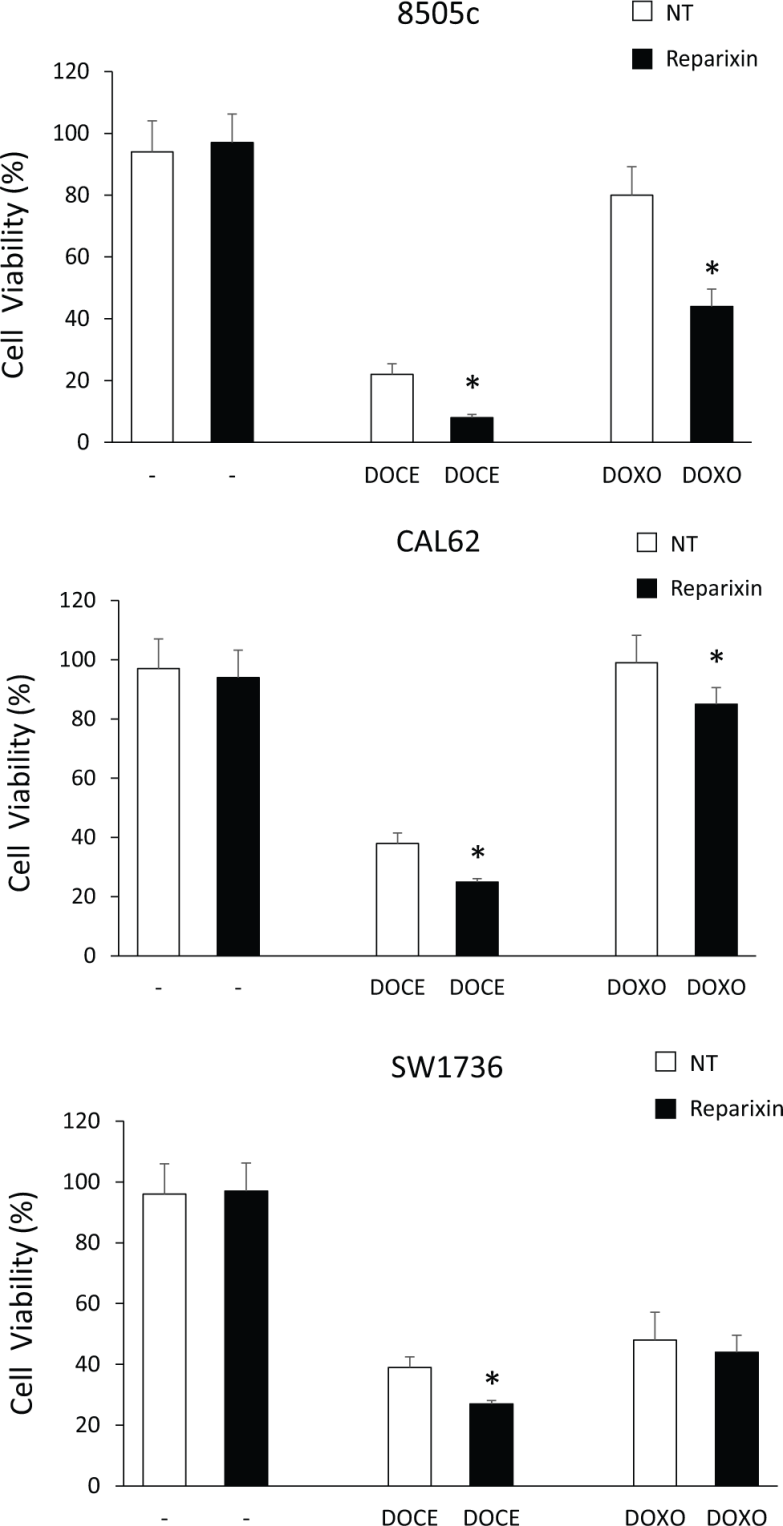
Reparixin potentiates anti-neoplastic effects of chemoterapics on TC cells Cell viability of 8505c, CAL62 and SW1736 cells left untreated (NT), or treated with Reparixin (10 μM), Docetaxel (DOCE-50 pM) or Doxorubicin (DOXO-5 nM) alone (white bars) or in combination (black bars) at 72 h. The percent (%) of live cells was reported. *p < 0.05 compared to the relative control.

Drug resistance is a major factor limiting the effectiveness of chemotherapy in many cancer types, including ATC [20], and has been linked to the expansion CSC compartment [6]. To investigate the possibility that Reparixin could sensitize ATC cells to chemotherapeutics commonly used in TC therapy, we plated 8505c, CAL62 and SW1736 in the presence or in the absence of suboptimal concentration of Reparixin (10 μM), in combination or not with suboptimal concentration of Docetaxel (DOCE-50 pM) or of Doxorubicin (DOXO-5 nM), and cell viability was evaluated after 72 h. Reparixin alone (10 μM) did not affect TC cell viability (Figure 7). Instead, Docetaxel (50 pM), although used at suboptimal concentrations, significantly affected ATC cell viability. Anyway, the combination of Reparixin with Docetaxel (50 pM) significantly reduced the percent of live cells compared to Docetaxel alone, indicating a significant synergic effect of the two drugs (Figure 7). Interestingly, Doxorubicin (5 nM) was not able to induce cell death in 8505c and in CAL62 cells. However, the addition of Reparixin to Doxorubicin, significantly reduced 8505c and CAL62 cell viability (Figure 7). The SW1736 cells displayed moderate sensitivity to Doxorubicin, and Reparixin treatment did not enhance their response to this chemotherapy (Figure 7).

These data suggest that Reparixin, depending on the cell context and on the drug used, could cooperate with conventional chemotherapics by enhancing their cytotoxic effects thus allowing their use at suboptimal concentrations.

### Reparixin inhibits TC cell tumorigenic activity

**Figure 8:**
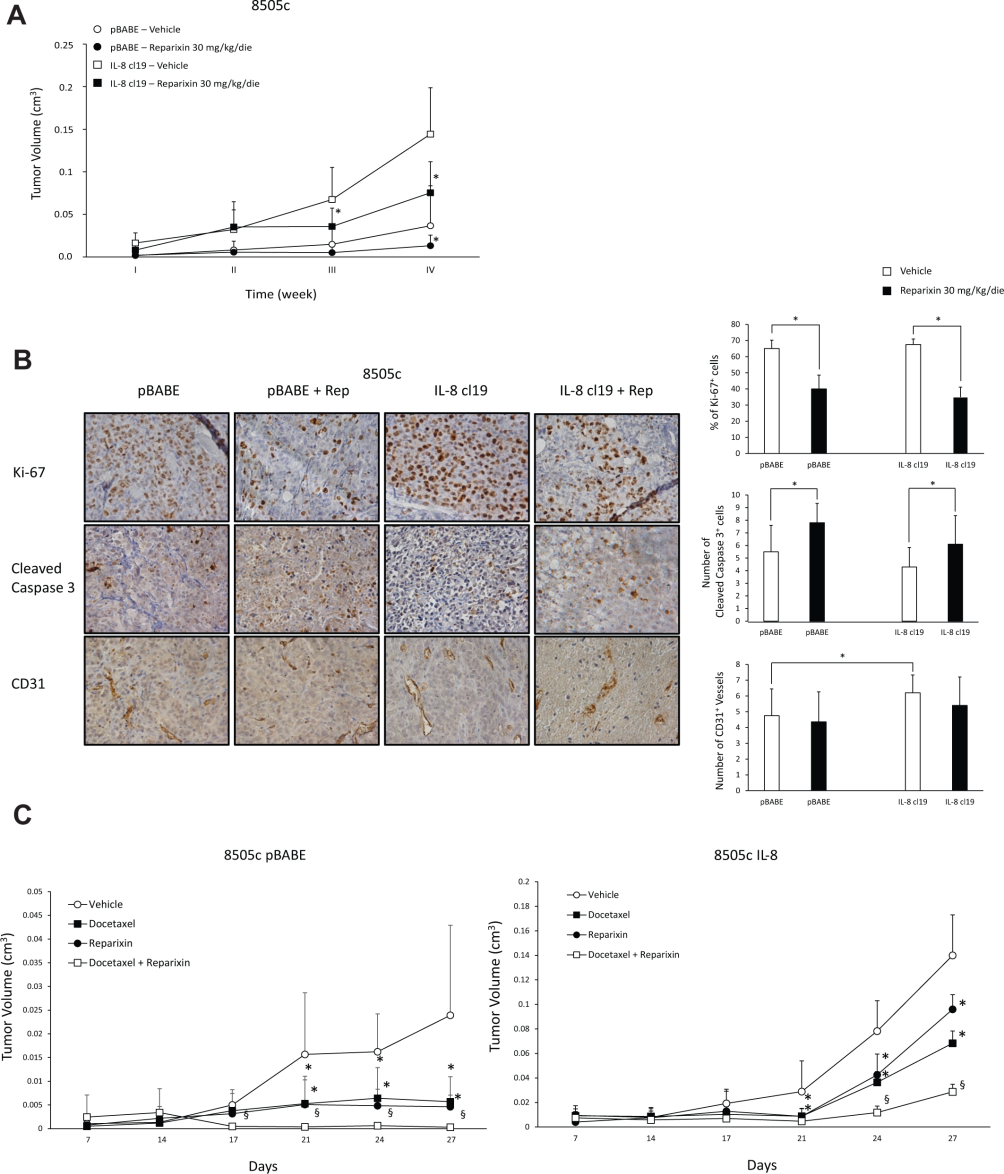
Reparixin inhibits xenograft growth of TC cells in nude mice (**A**) Growth curves of xenografts of 8505c cells stably overexpressing IL-8 (clone 19) or control cells (pBABE) in immunocompromised mice, treated with 30 mg/kg/die Reparixin or PBS vehicle. *P < 0.05 compared to the relative vehicle-treated group of mice. (**B**) IHC on excised IL-8 overexpressing or pBABE tumors treated or not with Reparixin for Ki-67^+^ cells, Cleaved caspase-3^+^ cells and CD31^+^ vessels (5 fields/sample). The quantification and representative photograms are shown. *p < 0.05 compared to relative vehicle-treated xenografts. (**C**) Effect of Docetaxel and Reparixin in 8505c pBABE (left panel) and IL-8 cl19 (right panel) xenografts. Docetaxel was injected i.p. at a dose of 5 mg/kg on days 9, 16, and 23; Reparixin was administred i.p. at 30 mg/Kg/die; combined treatment included both drugs. Control group mice received vehicle injections only. For 27 days, tumor size was monitored twice a week. *P < 0.05 compared to the relative vehicle-treated group of mice; ^§^*P* < 0.05 compared to the single drug-treated group of mice.

To define the effects of Reparixin on ATC cell tumorigenicity, we xenografted, into athymic (CD1 nu/nu) mice, control 8505c (8505c pBABE) or IL-8 ectopically expressing 8505c (8505c IL-8 cl19) cells (1 × 10^7^), that we previously generated [4]. Animals were randomized to receive intraperitoneal injection of vehicle (PBS) or Reparixin (30 mg/Kg/day), and tumor growth rate was monitored. The treatment was well tolerated, and we did not observe signs of general toxicity or body weight loss during the study. As already reported [4], 8505c IL-8-xenografted mice showed a significant increase in tumor growth rate compared to controls (Figure 8A). Importantly, Reparixin treatment caused a significant reduction of 8505c pBABE and 8505c IL-8 tumor volumes in comparison to vehicle-treated mice. In fact, at 4 weeks, 8505c pBABE tumor mean volume was 0.036 cm^3^, whereas 8505c pBABE xenografts in Reparixin-treated mice reached a mean volume of 0.013 cm^3^. Similarly, 8505c IL-8 tumor mean volume was 0.144 cm^3^, while Reparixin-treated 8505c IL-8 xenografts reached a mean volume of 0.075 cm^3^ (Figure 8A).

The effects of Reparixin on tumor growth were further investigated by analyzing excised tumors by immunohistochemistry (IHC). This analysis revealed that Reparixin significantly reduced proliferation and increased apoptotic rate of both 8505c pBABE and 8505c IL-8, when compared to the corresponding vehicle-treated xenografts (Figure 8B). 8505c IL-8 tumors exhibited a higher number of vessels compared to 8505c pBABE xenografts. Reparixin treatment decreased vessel density in 8505c IL-8 and, to a lesser extent, in 8505c pBABE xenografts, although these differences did not reach statistical significance at this stage (Figure 8B).

No effects of Reparixin on tumor incidence were observed in mice xenografted with 1 × 10^7^ 8505c pBABE or 8505c IL-8 cl19 cells. To better dissect the effect of the drug on the clonal capacity of TC cells *in vivo*, we performed a limiting dilution assay by injecting 8505c pBABE or IL-8 (1 × 10^6^, 1 × 10^5^, 1 × 10^4^ cells/mouse) subcutaneously in mice treated intraperitoneally with 30 mg/Kg/die of Reparixin or with vehicle (PBS). No tumor formation was observed in the groups of mice injected (10/group) with 1 × 10^5^, 1 × 10^4^ cells both in the presence and in the absence of Reparixin. However, when mice were injected with 1 × 10^6^ TC cells, we observed tumor formation, and Reparixin (30 mg/Kg/day) significantly reduced the incidence of both 8505c pBABE and 8505c IL-8 xenografts (Table 1).

**Table 1:** Tumor incidence (%) for pBABE and IL-8 cl19 8505c xenotransplants treated or not with Reparixin

Tumor Incidence (%)		
	Vehicle	Reparixin
8505c pBABE	66,7	10*
8505c IL-8 Cl19	90	50*

^*^ *P* < 0.05 compared to the relative vehicle-treated group of mice.

To evaluate also the ability of Reparixin to sensitize TC cells to chemotherapics, we xenografted 8505c pBABE or 8505c IL-8 cl19 (1 × 10^7^ cells) into athymic (CD1 nu/nu) mice randomized to receive intraperitoneal injection of vehicle (PBS), Reparixin (30 mg/Kg/day), Docetaxel (5 mg/kg once a week) or Reparixin and Docetaxel in combination. We observed a significant inhibition of 8505c pBABE and IL-8 tumor growth rates induced by treatment with Docetaxel or Reparixin, used as single drugs (Figure 8C). Moreover, the combination of Reparixin with Docetaxel significantly reduced tumor volume of both 8505c pBABE and IL-8 compared to the single agents (Figure 8C).

Taken together these results demonstrated that Reparixin exerts significant effects on TC tumor initiation and progression.

## DISCUSSION

Several human cancers hijack chemokine networks on their own benefit by exploiting autocrine signaling to increase their growth/viability and paracrine effects on infiltrating leukocytes to convert them into immune-tolerant, cancer-promoting cells [21]. In this context, several antibodies and small molecules have been generated against chemokines and their receptors that are in clinical trials for cancer therapy. The promising effects of these approaches are supported by the recent approval of a monoclonal antibody targeting CCR4 (mogamulizumab) for the treatment of adult T-cell leukemia [22] and by the results obtained with plerixafor (AMD3100), a blocking compound targeting CXCR4, able to enhance the sensitivity of tumor cells to cytotoxic agents by disrupting their interaction with the tumor microenvironment [23].

The up-regulation of the IL-8 receptors CXCR1 and CXCR2 is a frequent occurrence in human cancer and has been related to tumor progression [3]. IL-8 expression has been detected in several cancer types, including solid tumors (prostate, melanoma, colon, breast, stomach, pancreas, and liver) [24–32] and hematological malignancies (AML, CLL, Hodgkin's lymphoma) [32]. The importance of IL-8 circuit in sustaining cancer progression has been further supported by evidence demonstrating the dependence of the CSC compartment on IL-8-mediated signaling [33]. In the context of TC, we recently found that IL-8 is overexpressed compared to normal thyroid tissues, and its levels correlate with lymph-nodal metastases in human TC samples. Moreover, IL-8 binding to its receptors CXCR1 and CXCR2 concomitantly sustains TC cell growth, malignant features and efficiently stimulates stemness [8].

Reparixin was originally designed to target CXCR1 and CXCR2 on the surface of neutrophils to prevent their migration to sites of inflammation, and showed efficacy in several animal models of inflammatory disorders [34]. In the context of cancer, Reparixin was able to reduce *in vivo* the tumor-initiating ability of breast cancer cells by affecting the CSC population [17] and to sensitize cancer cells to chemotherapics [35]. Moreover, Reparixin alone or in combination with 5-Fluorouracil inhibited gastric cancer cell proliferation, survival, and migration both *in vitro* and *in vivo* [18].

Here, we provide evidence that Reparixin treatment affects various phenotypes of TC cells. Genetic depletion of both CXCR1 and CXCR2 affected proliferation, survival and motility of TC cells. Furthermore, the IL-8/CXCR1/CXCR2 signaling was crucial for the maintenance of stemness features and tumor-initiating ability of TC cells, whereas CXCL1/CXCR2 was not [8]. Consistently, here we provide evidence that Reparixin, by blocking both receptors, affects several functions of TC cells, including cell viability and EMT [4]. In accordance with our previous data demonstrating that the IL-8/CXCR1 circuit is enriched in the TC stem cell compartment [8], here we demonstrate that Reparixin significantly reduces the expression of stemness features. Importantly, Reparixin does not affect non-malignant thyroid epithelial cell growth and survival even at the highest concentrations, indicating that it exerts toxic effects preferentially on cancerous cells. This could be partially ascribed to the lower expression levels of CXCR1 and CXCR2 in thyroid normal compared to transformed cells [4, 8].

Reparixin single agent inhibits ATC xenograft growth and incidence in nude mice. We infer that the broad activity of Reparixin as a single agent in TC, at a variance with the breast cancer model, is due to the high expression of IL-8 receptors in TC [4] compared to the breast cancer cells [17], and to its ability to target both TC staminal and bulk cell populations. Treatment of mice with Reparixin significantly affects both cell proliferation and survival in xenotransplants. Furthermore, Reparixin appears to induce a decrease in vessel density, although without statistical significance, and a reduction of vessel calipers, that could contribute to the reduced proliferation and survival observed in Reparixin-treated xenografts. Mice lack the IL-8 functional gene, but express the murine orthologues of human GROα (CXCL1; i.e. murine KC) and GROβ (CXCL2; MIP2α), ligands of CXCR2 [36]. However, neither mouse KC nor CXCL2 exerts any effect on human TC cell proliferation and apoptosis (data not shown), indicating that the anti-neoplastic effects of Reparixin in xenografts are mainly due to the blockade of TC cell-derived IL-8.

Growing evidence demonstrated that increased chemokine expression by tumor cells modulates not only cancer development and/or progression but also resistance to chemotherapy [35, 37], even because it affects the CSC compartment [6]. In the present study, we demonstrate that the combination of Reparixin with Docetaxel or Doxorubicin results in a synergic effect on the reduction of cell viability and on tumor growth, consistently with data published in the breast cancer model [17, 35]. Interestingly, ATC is hardly treatable with conventional therapy [8], possibly because of its pronounced stemness features [19]. Thus, the synergic effects of Reparixin and classic chemotherapics may improve therapy of these resistant forms of TC.

Our results support, together with other reports, a role for IL-8 in TC aggressiveness [10, 11]. Thus, we can envisage that the inhibition of the IL-8 signaling pathway by using receptor antagonism (i.e., Reparixin) or other molecules inhibiting IL-8 secretion or activity [10, 11] might represents a new strategy for TC therapy both alone and in combination with chemotherapy.

## MATERIALS AND METHODS

### Cell cultures, transfection, sphere-forming assay, treatment with Reparixin

Human thyroid cell lines 8505c, CAL62, SW1736, Nthy and rat thyroid epithelial cells PC CL3 were maintained as previously described [8, 16]. For growth curves, cells were plated at a density of 0.5 × 10^5^ in complete medium (DMEM 10% FBS) and counted at the indicated time-points. Cell viability was assessed by Trypan blue staining. For spheroid-forming assays, cells were plated at 5 cells/well in ultra-low-attachment 96-well (Corning, NY, USA) in serum-free DMEM/F12 (1:1, Life Technologies), supplemented with 2% B27 and enriched with 10 ng/ml of Epidermal Growth Factor and 20 ng/ml of Basic Fibroblast Growth Factor (Miltenyi Biotec, Bergisch Gladbach, Germany). Spheres were counted after 15 days. For self-renewal assay, primary thyrospheres (F1) were collected by gentle centrifugation (1000 rpm/5 min) and dissociated mechanically, passed through 40 μm mesh filters (BD Falcon, Franklin Lakes, NJ) to eliminate aggregates and plated at 5 cells/well on ultra-low-attachment 96-well plates (Corning) to generate second (F2), third (F3) and fourth (F4) generations of thyrospheres. Quantification of sphere number was obtained by the formula (number of formed spheres/number of wells containing cells) × 100 [4]. In limiting dilution assays, cells were deposited at 1, 5, 10, and 50 cells/well. Clonal frequency and statistical significance were evaluated with the Extreme Limiting Dilution Analysis ‘limdil’ function (http://bioinf.wehi.edu.au/software/elda/index.html) [38].

Reparixin, kindly provided by Dompé Farmaceutici S.p.A., was prepared in PBS (pH 7.9-8.1). Treatment with IL-8 were performed using optimal concentration (100 ng/ml) as defined in several previous report [4, 8].

### S-phase entry

S-phase entry was evaluated by BrdU incorporation and indirect immunofluorescence. Cells were grown on coverslips, serum-deprived and treated with stimuli for 24 h. BrdU was added at a concentration of 10 μM for the last 30 min. BrdU-positive cells were revealed with Texas Red conjugated secondary Abs (Jackson ImmunoResearch Laboratories, West Grove, PA, USA) [39]. Cell nuclei were identified by Hoechst staining. Fluorescence was visualized with a Zeiss 140 epifluorescent microscope (Zeiss, Oberkochen, Germany).

### TUNEL assay

For the TUNEL assay, an equal number (5 × 10^3^) of cells was seeded onto single-well Costar glass slides (Corning Inc., Acton, MA, USA); cells were serum-deprived for 12 h, treated with different stimuli for 24 h and subjected to the TUNEL reaction (Roche, Basel, Switzerland) as described elsewhere [39].

### Cytofluorimetric analysis

For cell cycle analysis, cells were permeabilized with ice-cold 70% ethanol in phosphate-buffer saline, stored at −20°C over-night, resuspended in phosphate-buffered saline containing 50 μg/ml propidium iodide and 100 mg/ml RNase, incubated at 30°C for 30 min, and analyzed with a FACS Calibur cytofluorimeter using CellQuest software (BD Biosciences, Mississauga, ON, Canada).

For specific marker detection, cells were incubated (30 min at 4°C) with specific or isotype control antibodies (Abs), following cell permeabilization with the Cytofix/Cytoperm kit (BD Biosciences) [39]. Human SLUG, SNAIL and SOX2 Abs were from R&D Systems. A total of 10^4^ events for each sample were acquired in all analyses.

### RNA, cDNA and real-time-PCR

Total RNA was isolated and retrotranscribed as previously described [39]. Real-time quantitative PCR was performed as previously described [39]. Normalization was performed using β-actin and GAPDH mRNAs levels. Primers sequences are listed in Table 2.

**Table 2:** List of primers

SNAIL	F 5′ctctaggccctggctgctac 3′ R 5′gcctggcactggtacttctt 3′
SLUG	F 5′ccttcctggtcaagaagcat 3′ R 5′cacagtgatggggctgtatg 3′
ZEB1	F 5′ aactgctgggaggatgacac 3′ R 5′gtcctcttcaggtgcctcag 3′
TWIST1	F 5′ ccggagcctagatgtcattg 3′ R 5′ ggcctgtctgcgtttctctt 3′
OCT 3/4	F 5′agcaaaacccggaggagt 3′ R 5′ccacatcggcctgtgtatatc 3′
SOX 2	F 5′gcgaaccatctctgtggtct 3′ R 5′aaaatggaaagttgggatcg 3′
NANOG	F 5′tacctcagcctccagcagat 3′ R 5′ ttgctattcttcggccagtt 3′
ABCG2	F 5′tggatttacggctttgcagc 3 R 5′tcctgttgcattgagtcctgg 3′
ALDH	F 5′gcaactgaggaggagctctg 3′ R 5′ttcgattaaatcagccaacttgt 3′
β-actin	F 5′ tgcgtgacattaaggagaag 3′ R 5′ gctcgtagctcttctcca 3′
GAPDH	F 5′ gtccactggcgtcttcac 3′ R 5′ cttgaggctgttgtcatacttc 3′

### Migration assays

Migration assay was elicited using a Boyden chamber or a wound healing assay. For the Boyden chamber, we used a 48-well microchemotaxis chamber (NeuroProbe, Gaithersburg, MD, USA) and 8-μm-pore polycarbonate membranes (Nucleopore, Pleasanton, CA, USA) coated with 10 μg/ml fibronectin (Sigma-Aldrich) as described elsewhere [39]. For the wound healing assay, 8505c cells (2 × 10^4^ cells/well) were plated on Oris™ Cell Migration Assay 96-well plates (Platypus Technologies, Madison, WI, USA). Each well contained a silicone stopper that prevented cell attachment in the center region of the well. After allowing the cells to adhere to the surface for 6 h (37°C, 5% CO_2_), the stoppers were removed to reveal a uniform 2 mm diameter detection zone in the well into which cells could then migrate. The number of migrated cells were identified by using automated microscopy (Operetta high-content wide-field fluorescence imaging system, coupled to Harmony software, PerkinElmer, Waltham, MA, USA). Images of wells were acquired with the Operetta using a 10× high NA objective every 3 h up to 18 h in the brightfield channel with an exposure time of 50 ms [8].

### Tumorigenicity in immunocompromised mice

Each group of 8 mice (4-week-old CD1 nu/nu female) was inoculated subcutaneously with 8505c transfected with pBABE or IL-8 (1 × 10^7^ cells/mouse) treated intraperitoneally with 30 mg/Kg/die of Reparixin (twice a day) or with vehicle (PBS) alone. For *in vivo* limiting dilution assay 8505c transfected with pBABE or IL-8 were inoculated (1 × 10^7^, 1 × 10^6^, 1 × 10^5^, 1 × 10^4^ cells/mouse) subcutaneously in mice treated intraperitoneally with 30 mg/Kg/die of Reparixin or with vehicle (PBS) alone. For the xenotransplantation experiments evaluating the combined effects of Reparixin and Docetaxel: i) Docetaxel, diluted in phosphate-buffered saline/DMSO (ratio 1:1), was injected i.p. at a dose of 5 mg/kg on days 9, 16, and 23; ii) Reparixin was administred i.p. at 30 mg/Kg/die; iii) combined treatment mice were given both drugs; iv) control group mice received vehicle injections only. For 27 days, tumor size was monitored twice a week, and body weight, feeding behavior, and motor activity of each animal were monitored as indicators of general health [40].

Tumor diameters were measured at regular intervals with a caliper. Tumor volumes (V) were calculated with the formula: V = A × B^2^/2 (A = axial diameter; B = rotational diameter). This study was conducted according to Italian regulations for experimentation on animals. Paraffin-embedded tumors were analyzed by immunohistochemistry (IHC) with anti-Ki-67 antibody from Biocare Medical (Concord, CA, USA), anti-cleaved caspase 3 and anti-CD31 from R&D Systems (Minneapolis, MN, USA) [4].

### Statistical analysis

The results are expressed as the mean ± SEM of at least 3 experiments. Values from groups were compared by using the paired Student *t* test or the Duncan test. *P value* < 0.05 was considered statistically significant. The effects of Reparixin on tumor incidence of TC cells was conducted using χ^2^ test.
